# Shared versus independent tibial tunnels for lateral meniscus posterior root repair with ACL reconstruction: Comparable outcomes with shorter operative time in the shared tunnel technique at 2 years

**DOI:** 10.1002/jeo2.70687

**Published:** 2026-03-18

**Authors:** Ashwanth Narayan Balashanmugam, Sanjay Ayyakkannu Kasthuriranganathan, Vignesh Murali, Praveen Ravi, Suresh Perumal, Arumugam Sivaraman

**Affiliations:** ^1^ Department of Arthroscopy and Sports Medicine Centre for Sports Science, Sri Ramachandra Institute of Higher Education and Research, Porur Chennai India

**Keywords:** anterior cruciate ligament reconstruction, independent tibial tunnel, lateral meniscus posterior root repair, magnetic resonance imaging, shared tibial tunnel

## Abstract

**Purpose:**

Lateral meniscus posterior root (LMPR) tears frequently accompany anterior cruciate ligament (ACL) injuries and can compromise knee biomechanics and stability if left unrepaired. The primary research question was whether LMPR repair performed using a shared ACL tibial tunnel provides clinical, patient‐reported and magnetic resonance imaging (MRI) outcomes equivalent to those of an independent transtibial pullout technique.

**Methods:**

A retrospective comparative study included 50 patients who underwent arthroscopic ACL reconstruction with concomitant LMPR repair between June 2019 and June 2022. Twenty‐four patients underwent LMPR repair using a shared ACL tibial tunnel technique and 26 patients underwent repair using an independent transtibial pullout technique. All procedures were performed by a single surgeon, with a minimum follow‐up of 2 years. Clinical assessment included Lachman grade, pivot‐shift grade, International Knee Documentation Committee (IKDC) score and Lysholm knee score. MRI evaluation assessed LMPR healing and lateral meniscus extrusion (LME).

**Results:**

Mean operative time was significantly shorter in the shared tunnel group (73.50 ± 8.51 min) compared with the independent tunnel group (97.00 ± 8.39 min; *p* < 0.001). Significant improvements in clinical stability and patient‐reported outcome measures were observed in both groups at 2‐year follow‐up (*p* < 0.001 for all within‐group comparisons), with no significant differences between groups. MRI‐based LMPR healing rates were comparable (*p* = 0.231). Both groups demonstrated significant reductions in LME at follow‐up (*p* < 0.001), with no significant difference between techniques (*p* = 0.181).

**Conclusion:**

LMPR repair using a shared ACL tibial tunnel yields clinical, patient‐reported and MRI outcomes comparable to those of an independent transtibial pullout technique, while significantly reducing operative time. By avoiding an additional tibial tunnel, this technique preserves bone stock, prevents tunnel convergence and eliminates the need for an extra implant, making it a safe, efficient and cost‐effective option when combined with arthroscopic ACL reconstruction.

**Level of Evidence:**

Level III, retrospective comparative study.

**Abbreviations**
ACL: anterior cruciate ligament
CI: confidence interval
ICC: intraclass correlation coefficient
IKDC: International Knee Documentation Committee
LME: lateral meniscus extrusion
LMPR: lateral meniscus posterior root
mm: millimetres
MRI: magnetic resonance imaging
PDFS: proton density fat‐suppressed
ROM: range of motion
STG: semitendinosus–gracilis

## INTRODUCTION

Lateral meniscal root tears are frequently associated with anterior cruciate ligament (ACL) injuries, with reported incidences ranging from 12% to 17% in ACL‐deficient knees [16, 17, 18]. The lateral meniscus posterior root (LMPR) plays a critical role in converting axial loads into circumferential hoop stresses, thereby maintaining normal tibiofemoral contact mechanics. Disruption of the LMPR leads to a reduction in contact area and an increase in mean and peak contact pressures in the lateral compartment, particularly during deep knee flexion and pivoting activities [1, 7, 11].

Beyond load transmission, the LMPR serves as an important secondary stabilizer of the knee. Biomechanical studies have demonstrated that LMPR deficiency significantly increases anterior tibial translation and rotational laxity, even in the presence of an intact ACL [8, 12]. Clinical investigations have further shown that unrepaired LMPR tears at the time of ACL reconstruction are associated with persistent instability, inferior functional outcomes and increased risk of early degenerative changes [6, 17]. Consequently, repair of LMPR tears during ACL reconstruction has gained widespread acceptance as a means to restore knee biomechanics and improve postoperative stability.

Transtibial pullout repair remains one of the most commonly employed techniques for LMPR fixation. Traditionally, this technique involves the creation of an independent tibial tunnel aimed at the anatomic footprint of the LMPR. While anatomic tunnel placement has been shown to restore near‐normal contact pressures and hoop stresses in biomechanical models [9, 14], the close spatial relationship between the LMPR and ACL tibial footprints poses a significant technical challenge when both tunnels are drilled independently. Recent cadaveric and imaging studies have reported tunnel convergence rates of approximately 20%–25% during simultaneous ACL reconstruction and LMPR repair, which may compromise graft fixation, biological healing or both [2, 5, 11].

To mitigate the risk of tunnel coalition, a shared ACL tibial tunnel technique has been proposed, whereby meniscal sutures are passed through the ACL tibial tunnel, eliminating the need for an additional bone tunnel. Anatomical studies have demonstrated that the ACL tibial footprint is located approximately 5–6 mm anterior to the native LMPR footprint, resulting in a non‐anatomic tibial suture exit point when a shared tunnel is used [3]. Despite this difference, anatomical reduction of the meniscal root to its native insertion site can still be achieved using transtibial sutures, raising the possibility that accurate reduction and stable fixation may be more critical to clinical success than the precise location of the tibial tunnel itself.

Early clinical studies evaluating shared tunnel techniques have reported favourable short to mid‐term outcomes, including significant improvements in patient‐reported outcome measures and knee stability, while also demonstrating reduced operative time and avoidance of tunnel overlap [15, 19, 20]. However, direct comparative studies evaluating shared versus independent tibial tunnel techniques for LMPR repair performed in conjunction with ACL reconstruction remain limited, particularly with respect to radiological healing and meniscal extrusion on magnetic resonance imaging (MRI).

The purpose of this study was therefore to compare the clinical, patient‐reported and MRI outcomes of LMPR repair performed using a shared ACL tibial tunnel versus an independent transtibial pullout tunnel, both in combination with arthroscopic ACL reconstruction, at a minimum follow‐up of 2 years. It was hypothesized that the shared tunnel technique would result in clinical and radiological outcomes comparable to those of the independent tunnel technique, while offering the advantage of reduced operative time and avoidance of tunnel coalition.

## METHODS

A retrospective study was conducted in which medical records of patients who underwent arthroscopic ACL reconstruction with concomitant LMPR repair between June 2019 and June 2022 were reviewed. All procedures were performed by a single surgeon. Of the 78 patients initially identified, 50 met the predefined inclusion and exclusion criteria and were included in the final analysis. Among these, 24 patients underwent LMPR repair using a shared ACL tibial tunnel technique, while 26 patients underwent LMPR repair using an independent transtibial pullout technique, both in combination with arthroscopic ACL reconstruction.

### Inclusion criteria

1. Skeletally mature patients who underwent ACL reconstruction using a semitendinosus–gracilis (STG) autograft.
2. Concomitant LMPR repair was performed using either a shared ACL tibial tunnel technique or an independent transtibial pullout repair.
3. Minimum clinical and radiological follow‐up of 2 years.

### Exclusion criteria

1. Associated posterior cruciate ligament injury or collateral ligament injuries (medial or lateral).
2. Chondral lesions greater than Grade II according to the Modified Outerbridge classification.
3. Revision ACL reconstruction procedures.
4. Severe coronal plane deformities requiring corrective osteotomy.
5. Patients who sustained a new knee injury during the follow‐up period.

### Surgical technique of shared tunnel

Under general anaesthesia, the patient was positioned supine with the legs hanging freely. A pneumatic tourniquet was applied and standard high anterolateral and anteromedial portals were established. Diagnostic arthroscopy was performed to confirm the presence of an ACL tear (Figure 1a) and a LMPR tear (Figure 1b).

**Figure 1:**
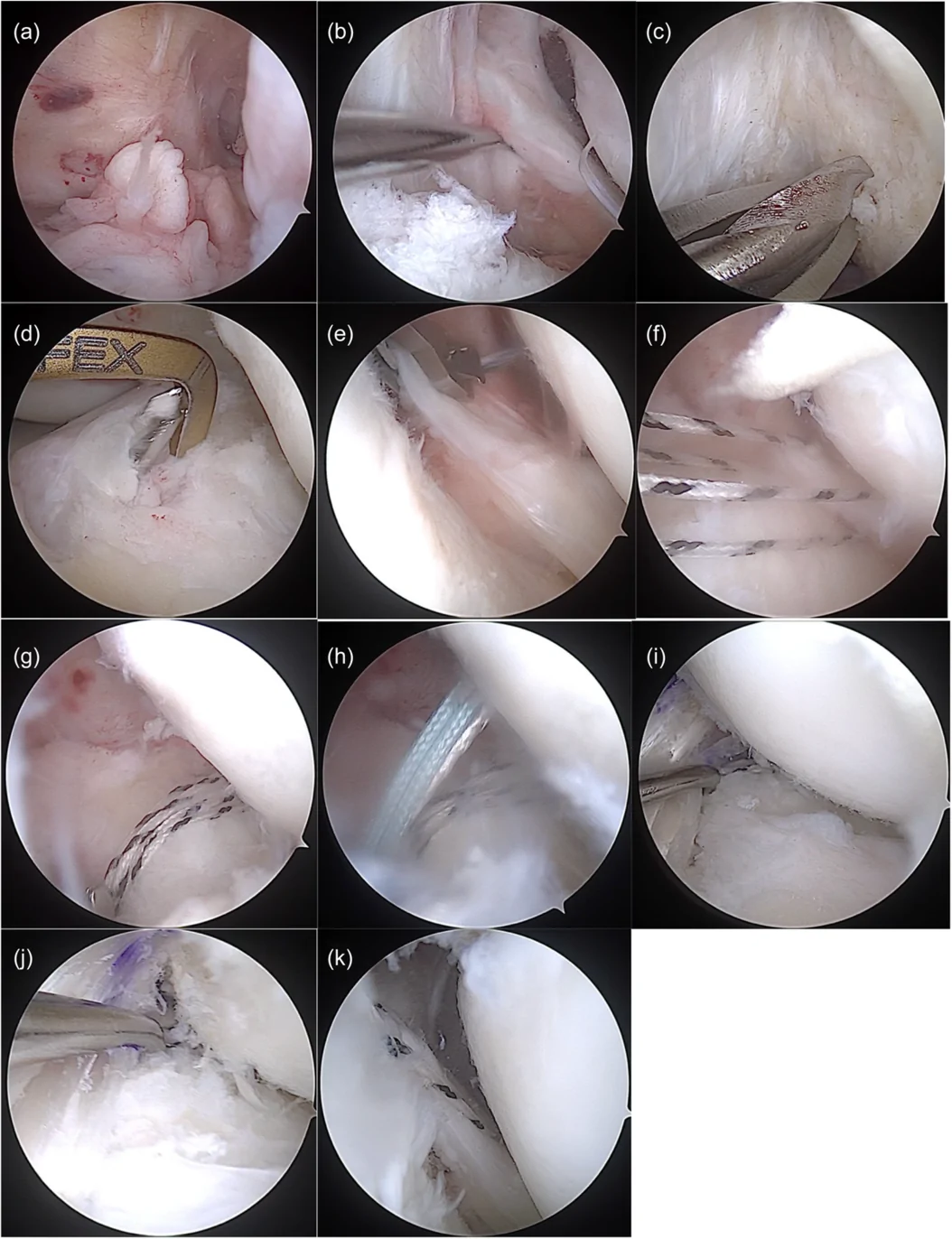
Surgical steps of lateral meniscus posterior root repair using the shared ACL tibial tunnel technique during arthroscopic ACL reconstruction. (a) Arthroscopic view demonstrating an anterior cruciate ligament (ACL) tear. (b) Arthroscopic view demonstrating a lateral meniscus posterior root (LMPR) tear. (c) Femoral tunnel drilled at the anatomic ACL footprint. (d) Tibial tunnel drilled at the anatomic ACL tibial footprint. (e) Passage of FiberLink sutures through the LMPR using a suture passer. (f) FiberLink sutures exchanged for two No. 2 FiberWire sutures. (g) Retrieval of LMPR sutures through the ACL tibial tunnel. (h) Passage of the ACL graft with LMPR sutures positioned posterior to the graft. (i) Anatomical reduction of the LMPR at its native insertion site confirmed arthroscopically. (j) Arthroscopic probing demonstrating stable fixation of the ACL graft. (k) Arthroscopic probing confirming stable fixation of the repaired LMPR. ACL, anterior cruciate ligament.

A STG autograft was harvested, prepared and soaked in vancomycin. The mean graft diameter was 8.2 ± 0.4 mm (range, 7.5–9.0 mm). The femoral tunnel (Figure 1c) was drilled at the anatomic ACL footprint, and the tibial tunnel (Figure 1d) was drilled at the anatomic ACL tibial footprint. Both femoral and tibial tunnels were created to match the measured graft diameter in each case.

The LMPR insertion site was debrided and curetted to enhance the biological environment and promote soft tissue‐to‐bone healing. Two sutures were passed through the LMPR using two FiberLink sutures and a suture passer (Figure 1e), which were subsequently exchanged for two No. 2 FiberWire sutures (Figure 1f). The sutures were retrieved through the ACL tibial tunnel (Figure 1g).

The ACL graft was then passed ensuring that the meniscal sutures were positioned posterior to the graft (Figure 1h) and fixed on the femoral side using suspensory fixation with a fixed‐loop device. Using controlled tension on the meniscal sutures and arthroscopic probing, the LMPR was anatomically reduced to its native insertion site (Figure 1i).

Tibial fixation of both the ACL graft and the meniscal sutures was performed using an interference screw with the knee positioned at 30° of flexion. To prevent abrasion of the meniscal sutures, the interference screw was placed anterior to the graft, thereby interposing the graft between the screw and the meniscal sutures. Additional extracortical fixation of the meniscal sutures was not required, as stable fixation was achieved with interference screw fixation alone. Final arthroscopic probing confirmed adequate tension and stability of both the ACL graft (Figure 1j) and the repaired LMPR (Figure 1k).

### Surgical technique of independent tunnel

Following confirmation of an ACL tear (Figure 2a) and a LMPR tear (Figure 2b) during diagnostic arthroscopy, the femoral tunnel was created at the anatomic ACL footprint (Figure 2c). A STG autograft was used in all cases, with a mean graft diameter of 8.2 ± 0.4 mm (range, 7.5–9.0 mm). Both femoral and tibial tunnels were drilled to match the measured graft diameter in each patient.

**Figure 2:**
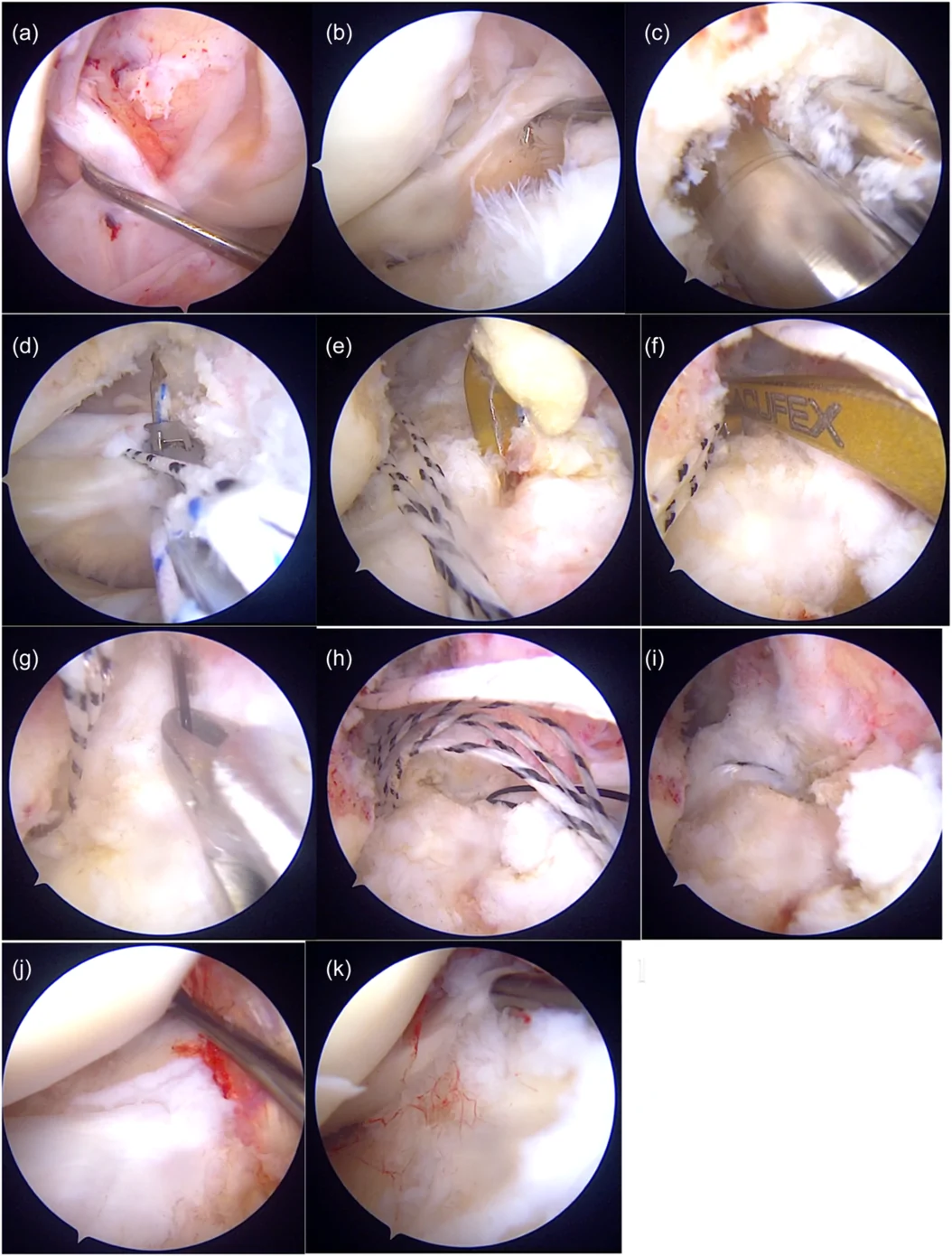
Surgical steps of lateral meniscus posterior root repair using the independent transtibial pullout technique during arthroscopic ACL reconstruction. (a) Arthroscopic view demonstrating an anterior cruciate ligament (ACL) tear. (b) Arthroscopic view demonstrating a lateral meniscus posterior root (LMPR) tear. (c) Femoral tunnel created at the anatomic ACL footprint. (d) Passage of FiberLink sutures through the LMPR using a suture passer. (e) Creation of the ACL tibial tunnel at the anatomic ACL tibial footprint using an ACL tibial jig set at 50°. (f) Creation of an independent tibial tunnel directed toward the anatomic LMPR footprint using an ACL tibial jig set at 60°. (g) Shuttle suture (No. 1 loop Ethilon) passed through the LMPR tibial tunnel. (h) FiberWire sutures shuttled through the LMPR tibial tunnel. (i) Reduction of the LMPR to its anatomic insertion site through the transtibial tunnel. (j) Arthroscopic probing confirming stable fixation of the ACL graft. (k) Arthroscopic probing demonstrating stable fixation of the repaired LMPR. ACL, anterior cruciate ligament.

The LMPR insertion site was prepared and two sutures were passed through the LMPR using two FiberLink sutures and a suture passer (Figure 2d), which were subsequently exchanged for two No. 2 FiberWire sutures. The ACL tibial tunnel was created at the anatomic ACL tibial footprint using an ACL tibial jig set at 50° (Figure 2e).

A separate tibial tunnel for the LMPR was then created using an ACL tibial jig set at 60°, aimed at the anatomic LMPR footprint (Figure 2f), with the tibial entry point positioned inferior and lateral to the ACL tibial tunnel. A guide pin was first advanced, followed by reaming with a 4.5‐mm cannulated reamer to create the LMPR tibial tunnel. A No. 1 loop Ethilon suture was used to shuttle the two FiberWire sutures through the LMPR tibial tunnel (Figure 2g,h) and the LMPR was reduced through the tunnel (Figure 2i).

The ACL graft was then passed through the femoral and tibial tunnels and fixed on the femoral side using suspensory fixation with a fixed‐loop device, followed by tibial fixation using an interference screw with the knee positioned at 30° of flexion. The meniscal sutures were subsequently tensioned and secured to the tibia using extracortical suspensory fixation. Final arthroscopic probing confirmed appropriate tension and stability of the ACL graft (Figure 2j) and the repaired LMPR (Figure 2k).

### Postoperative rehabilitation

Active calf stretching exercises were initiated on the day of surgery. Isometric quadriceps and hamstring co‐contraction exercises, patellar mobilization and straight leg raises in two planes were commenced on the first postoperative day. Weight bearing as tolerated was allowed from the first postoperative day with the use of a long knee brace (non‐range‐of‐motion [ROM] brace).

The knee was maintained in full extension using the long knee brace for a minimum of 10 days to prevent fixed flexion deformity. Following suture removal, ROM exercises were initiated. Full weight bearing without the knee brace was permitted at the end of 4 weeks.

Full knee ROM was achieved by 6 weeks postoperatively, after which strengthening exercises were progressively intensified. Low‐impact aerobic activities, including jogging, swimming and cycling, were initiated at approximately 3 months. Return to non‐contact sports was permitted after 6 months, while return to contact or pivoting sports was allowed after 8 months following surgery.

### Assessment parameters

Age, sex, body mass index, affected side, mode of injury, time from injury to surgery (Table 1) and duration of surgery were recorded for all patients. Clinical assessment included evaluation of Lachman grade, pivot‐shift grade, International Knee Documentation Committee (IKDC) score and Lysholm knee score, which were documented preoperatively and at the 2‐year follow‐up (Table 2).

**Table 1:** Baseline demographic and clinical characteristics of the study population.

Parameters	Group 1 (shared tunnel) (*n* = 24)	Group 2 (independent tunnel) (*n* = 26)	Inter‐group comparison, *p* value
Age (years) (mean ± SD)	26.50 ± 4.791	28.69 ± 7.030	0.207
Sex, *n* (%) [M/F]	19(79.1)/5(20.9)	20(76.9)/6(23.1)	0.848
BMI	25.10 ± 4.970	25.22 ± 5.277	0.935
Affected side, *n* (%) [R/L]	12(50)/12(50)	15(57.7)/11(42.3)	0.586
Mode of injury in *n* (%) (sport/fall/motor vehicle accident)	10(41.7)/5 (20.8)/9(37.5)	11(42.3)/6(23.1)/9(34.6)	0.971
Time (months) from injury to surgery in median (interquartile range)	5(3.25, 7)	4.5(2, 8.25)	0.822

Abbreviations: BMI, body mass index; F, female; L, left; M, male; R, right; SD, standard deviation.

**Table 2:** Clinical outcomes and patient‐reported outcome measures were compared between groups and within groups at preoperative assessment and at 2‐year follow‐up.

	Group 1—Shared tunnel (*n* = 24)	Group 2—Independent tunnel (*n* = 26)	Inter‐group comparison, *p* value
Lachman grade in *n* (%) (Grade 0/1/2/3)			
Pre‐operative	0(0)/0(0)/2(8.3)/22(91.7)	0(0)/0(0)/5(19.2)/21(80.8)	0.267
2‐year follow‐up	23(95.8)/1(4.2)/0(0)/0(0)	24(92.3)/2(7.7)/0(0)/0(0)	0.600
Pre‐operative versus follow‐up comparison, *p* value	<0.001	<0.001	
Pivot shift grade in *n* (%) (Grade 0/1/2/3)			
Pre‐operative	0(0)/3(12.5)/15(62.5)/6(25)	0(0)/3(11.5)/19(73.1)/4(15.4)	0.673
2‐year follow‐up	23(95.8)/1(4.2)/0(0)/0(0)	25(96.2)/1(3.8)/0(0)/0(0)	0.954
Pre‐operative versus follow‐up comparison, *p* value	<0.001	<0.001	
Lysholm knee score (mean ± standard deviation)			
Pre‐operative	45.00 ± 8.325	45.77 ± 9.240	0.759
2‐year follow‐up	91.50 ± 3.007	91.35 ± 3.032	0.858
Pre‐operative versus follow‐up comparison, *p* value	<0.001	<0.001	
Lysholm knee score improvement (mean ± standard deviation)	46.50 ± 8.166	45.57 ± 9.944	0.723
IKDC score (mean ± standard deviation)			
Pre‐operative	44.23 ± 9.403	41.41 ± 11.573	0.936
2‐year follow‐up	90.52 ± 2.718	89.67 ± 3.067	1.029
Pre‐operative versus follow‐up comparison, *p* value	<0.001	<0.001	
IKDC knee score improvement (mean ± standard deviation)	46.29 ± 8.511	48.26 ± 10.079	0.464

Abbreviation: IKDC, International Knee Documentation Committee.

Preoperative MRI was reviewed to confirm the presence of a LMPR tear, assess meniscal extrusion, identify ACL injury and evaluate associated chondral lesions and other ligament injuries. MRI performed at the 2‐year follow‐up was assessed for LMPR healing status and lateral meniscus extrusion (LME) (Table 3).

**Table 3:** Lateral meniscus extrusion was compared between groups and within groups at preoperative assessment and at 2‐year follow‐up.

Lateral meniscus extrusion (mm) (mean ± standard deviation)	Group 1—Shared tunnel (*n* = 24)	Group 2—Independent tunnel (*n* = 26)	Inter‐group comparison, *p* value	95% confidence interval of the difference	
Pre‐operative	3.38 ± 0.129	3.49 ± 0.181	0.017		
2‐year follow‐up	1.24 ± 0.284	1.48 ± 0.254	0.002		
Pre‐operative versus follow‐up comparison, *p* value	<0.001	<0.001			
Lateral meniscus extrusion reduction (mm)	2.14 ± 0.347	2.01 ± 0.352	0.181	**Lower** −0.064	**Upper** 0.334

LMPR healing was evaluated on T2‐weighted MRI sequences and categorized into three grades:

Grade 1 (Complete healing): Normal low‐intensity signal within the LMPR (Figure 3).

**Figure 3:**
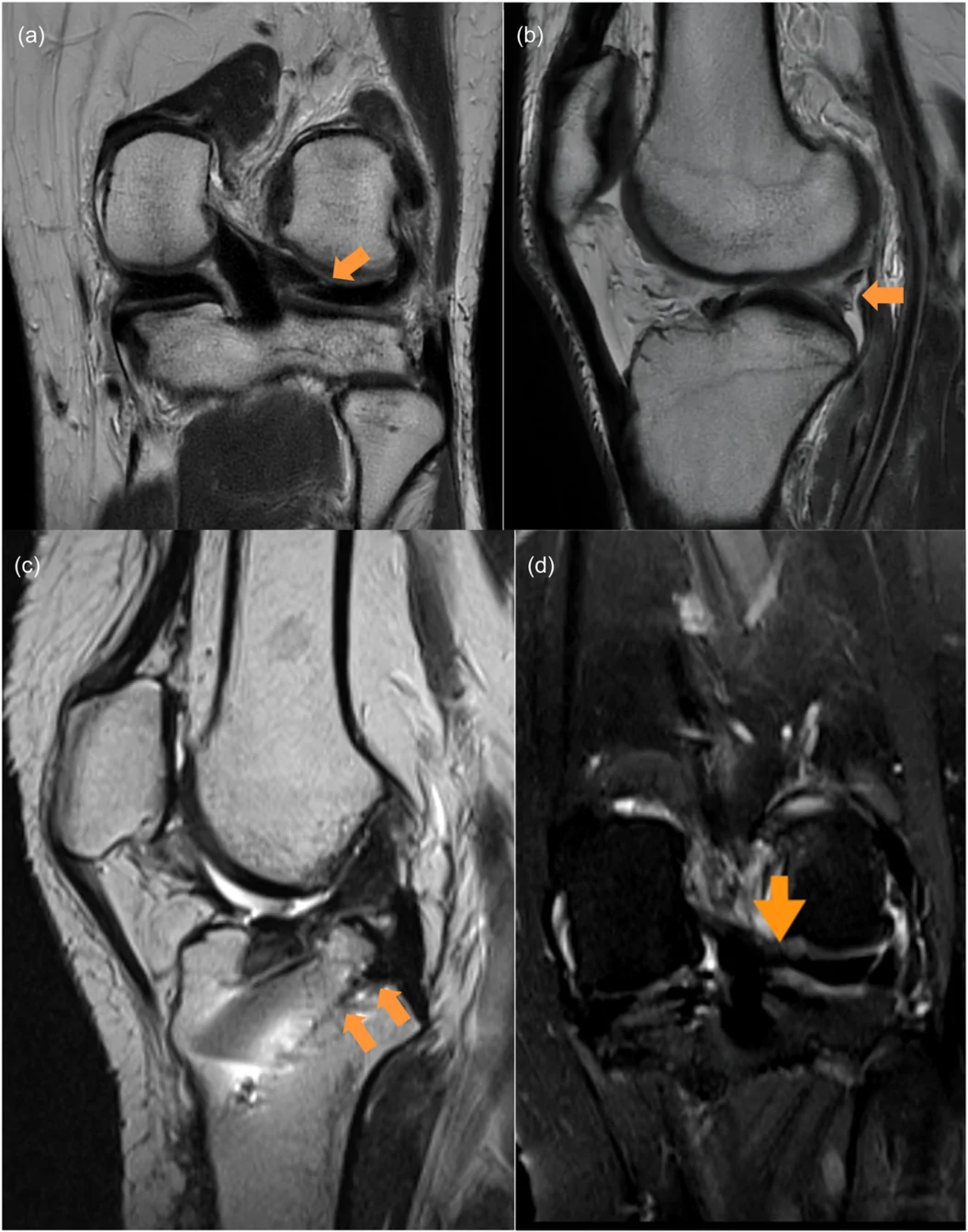
Magnetic resonance imaging (MRI) examples illustrating complete healing of the lateral meniscus posterior root (LMPR) following repair using the independent transtibial pullout technique. (a) Preoperative T2‐weighted coronal image demonstrating a tear extending into the LMPR. (b) Preoperative T2‐weighted sagittal image demonstrating the “ghost sign” consistent with an LMPR tear. (c) Two‐year follow‐up T2‐weighted sagittal image demonstrating complete LMPR healing. (d) Two‐year follow‐up proton density fat‐suppressed (PDFS) coronal image of the same patient confirming complete LMPR healing.

Grade 2 (Partial healing): High‐intensity signal involving more than one‐third, but not the entire LMPR (Figure 4).

**Figure 4:**
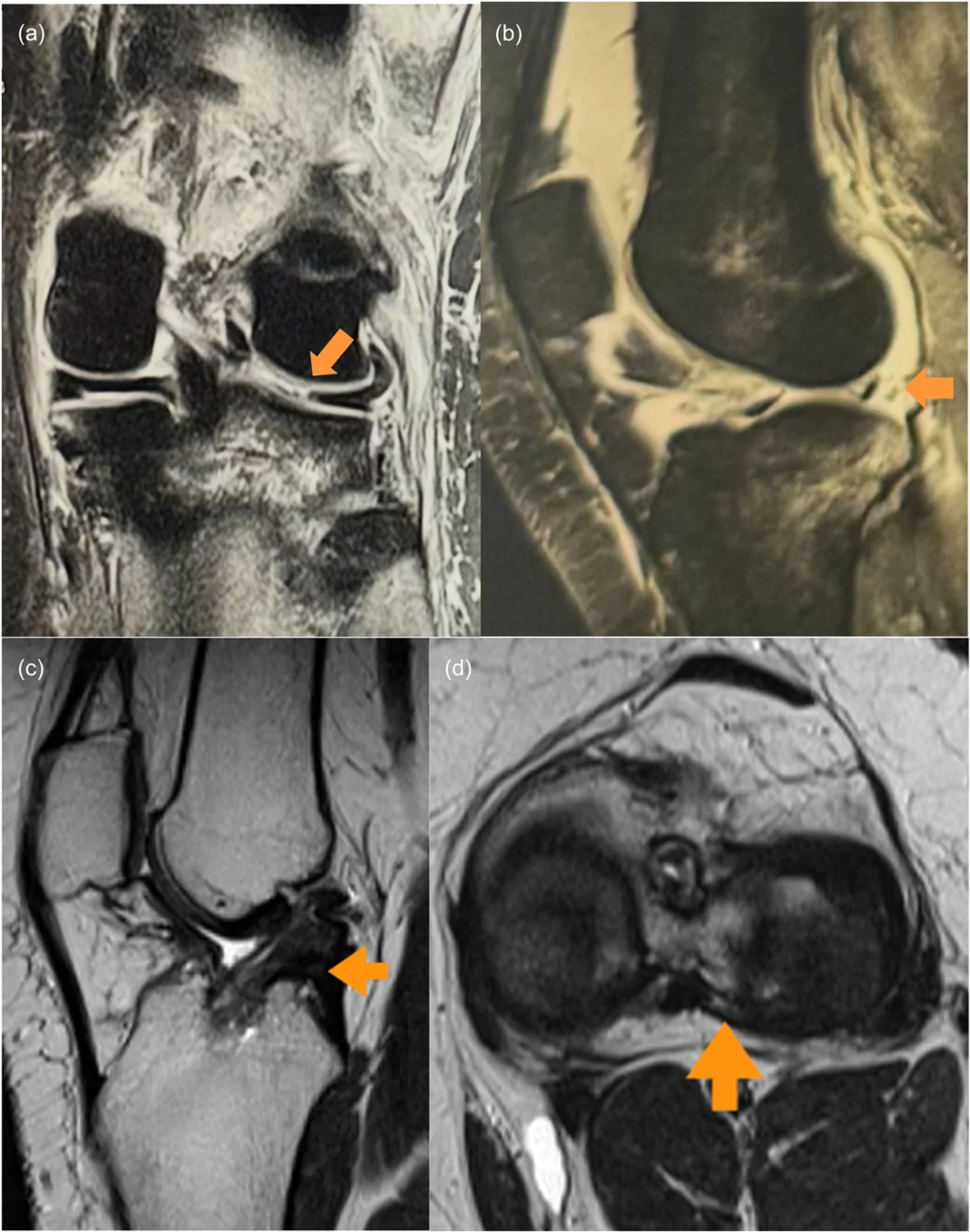
Magnetic resonance imaging (MRI) images demonstrating partial healing of the lateral meniscus posterior root (LMPR) following repair using the shared ACL tibial tunnel technique. (a) Preoperative proton density fat‐suppressed (PDFS) coronal image demonstrating a tear extending into the LMPR. (b) Preoperative PDFS sagittal image demonstrating the characteristic ‘ghost sign’ of an LMPR tear. (c) Two‐year follow‐up T2‐weighted sagittal image demonstrating partial LMPR healing. (d) Two‐year follow‐up T2‐weighted axial image of the same patient further demonstrating partial LMPR healing. ACL, anterior cruciate ligament.

Grade 3 (Failed healing): Replacement of the entire LMPR by a high‐intensity signal [20].

LME was measured in millimetres (mm) on coronal MRI sequences at the level corresponding to the midpoint of the lateral femoral condyle, as identified on the sagittal plane. The extent of extrusion was defined as the horizontal distance from the lateral edge of the lateral tibial plateau to the outermost margin of the lateral meniscus, as previously described [4, 10] (Figure 5).

**Figure 5:**
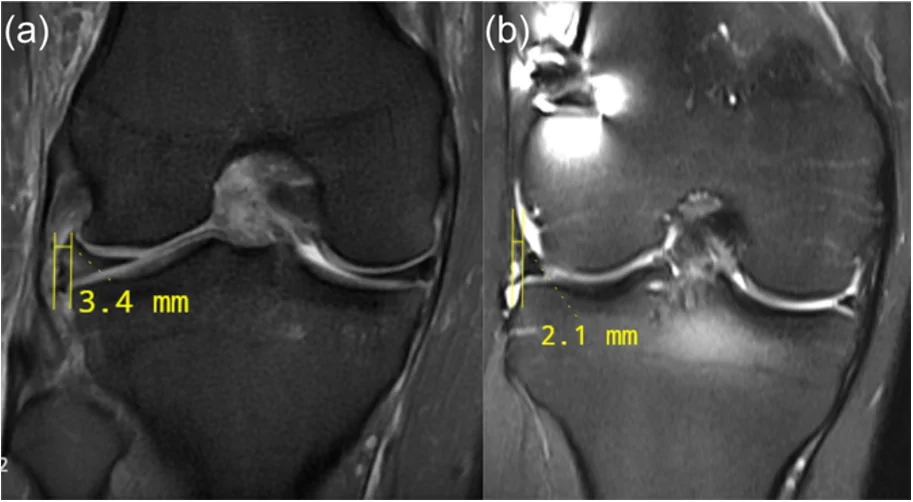
Magnetic resonance imaging (MRI) demonstrating the method used for measurement of lateral meniscus extrusion (LME). (a) Preoperative proton density fat‐suppressed (PDFS) coronal image showing measurement of LME. (b) Two‐year follow‐up PDFS coronal image of the same patient demonstrating reduction in LME following LMPR repair. LMPR, lateral meniscus posterior root.

MRI evaluations were independently performed by two musculoskeletal radiologists who were blinded to the clinical data and surgical technique. A standardized scoring system was used and both reviewers underwent calibration training prior to image assessment.

For meniscal root healing, intra‐observer reliability was excellent, with an intraclass correlation coefficient (ICC) of 0.91 (95% confidence interval [CI], 0.879–0.942) and interobserver reliability was also excellent, with an ICC of 0.90 (95% CI, 0.870–0.935).

For LME measurements, interobserver reliability demonstrated excellent agreement, with an ICC of 0.88 (95% CI, 0.840–0.920). Intra‐observer reliability for LME was likewise excellent, with an ICC of 0.93 (95% CI, 0.900–0.960).

### Statistical analysis

A post hoc sample size and power analysis was performed to assess whether the study was adequately powered to detect clinically meaningful differences between the two groups. Based on previously published literature evaluating clinical outcomes following LMPR repair performed in conjunction with ACL reconstruction, a minimum clinically important difference of 8 points for the IKDC score was considered relevant, with an assumed standard deviation of 10 points. Using a two‐tailed independent samples *t* test, an *α* level of 0.05 and a desired power of 80%, a minimum sample size of 23 patients per group was required. The final study cohort included 24 patients in the shared tunnel group and 26 patients in the independent tunnel group, thereby meeting the calculated sample size requirement.

Statistical analysis was performed using SPSS software (Version 19; IBM Corp.). Quantitative variables, including age, body mass index, duration of surgery, Lysholm knee score, IKDC score, LME and time to return to sports were expressed as mean (standard deviation). Time from injury to surgery was reported as median (interquartile range). Qualitative variables, including sex, affected side, mode of injury, Lachman grade, pivot‐shift grade, LMPR healing and return to pre‐injury level of sports were presented as frequencies and percentages.

Normality of data distribution was assessed using the Shapiro–Wilk test. Categorical variables were analysed using the *χ* ^2^ test. Comparisons of normally distributed continuous variables between the two independent groups were performed using the independent samples *t* test, while within‐group comparisons (preoperative versus follow‐up) were analysed using the paired *t* test. For variables that did not follow a normal distribution, the Mann–Whitney *U* test was used for intergroup comparisons and the Wilcoxon signed‐rank test for intragroup comparisons. All statistical tests were two‐tailed and a *p* value of less than 0.05 was considered statistically significant.

## RESULTS

### Patient demographics and follow‐up

A total of 50 patients met the inclusion criteria and were included in the final analysis, comprising 24 patients in the shared ACL tibial tunnel group and 26 patients in the independent transtibial pullout repair group. Baseline demographic characteristics, including age, sex distribution, body mass index, affected side, mode of injury and time from injury to surgery, were comparable between the two groups (Table 1). A minimum follow‐up of 24 months was completed by all patients.

### Operative time

The mean duration of surgery was significantly shorter in the shared tunnel group (73.50 ± 8.51 min) compared with the independent tunnel group (97.00 ± 8.39 min) (*p* < 0.001).

### Clinical outcomes

Lachman grade, pivot‐shift grade, IKDC score and Lysholm knee score demonstrated significant improvement from the preoperative assessment to the 2‐year follow‐up in both groups (*p* < 0.001 for all parameters).

In the shared tunnel group, the mean IKDC score improved from 44.23 ± 9.40 preoperatively to 90.52 ± 2.72 at 2‐year follow‐up, while the Lysholm knee score improved from 45.00 ± 8.33 to 91.50 ± 3.01. In the independent tunnel group, the mean IKDC score improved from 41.41 ± 11.57 to 89.67 ± 3.07 and the Lysholm knee score improved from 45.77 ± 9.24 to 91.35 ± 3.03 at follow‐up. The magnitude of improvement in IKDC and Lysholm knee scores did not differ significantly between the two groups (*p* = 0.464 and *p* = 0.723, respectively).

At final follow‐up, the majority of patients in both groups demonstrated a negative Lachman and pivot‐shift test, with no statistically significant differences between groups (Table 2).

### Radiological outcomes

MRI evaluation at the 2‐year follow‐up was available for all patients (shared tunnel group, *n* = 24; independent tunnel group, *n* = 26).

LME demonstrated significant reduction within both groups. In the shared tunnel group, mean LME decreased from 3.38 ± 0.13 mm preoperatively to 1.24 ± 0.28 mm at follow‐up (*p* < 0.001). In the independent tunnel group, LME decreased from 3.49 ± 0.18 to 1.48 ± 0.25 mm (*p* < 0.001). The magnitude of LME reduction was greater in the shared tunnel group (2.14 ± 0.35 mm) compared with the independent tunnel group (2.01 ± 0.35 mm); however, this difference was not statistically significant (*p* = 0.181).

Regarding LMPR healing on MRI at 2‐year follow‐up, healing grades were comparable between the two groups (*p* = 0.231). In the shared tunnel group (*n* = 24), 16 patients (66.7%) demonstrated complete healing, 8 patients (33.3%) demonstrated partial healing and 0 patients (0%) demonstrated failed healing. In the independent tunnel group (*n* = 26), 21 patients (80.8%) demonstrated complete healing, 4 patients (15.4%) demonstrated partial healing and 1 patient (3.8%) demonstrated failed healing (Figure 6).

**Figure 6:**
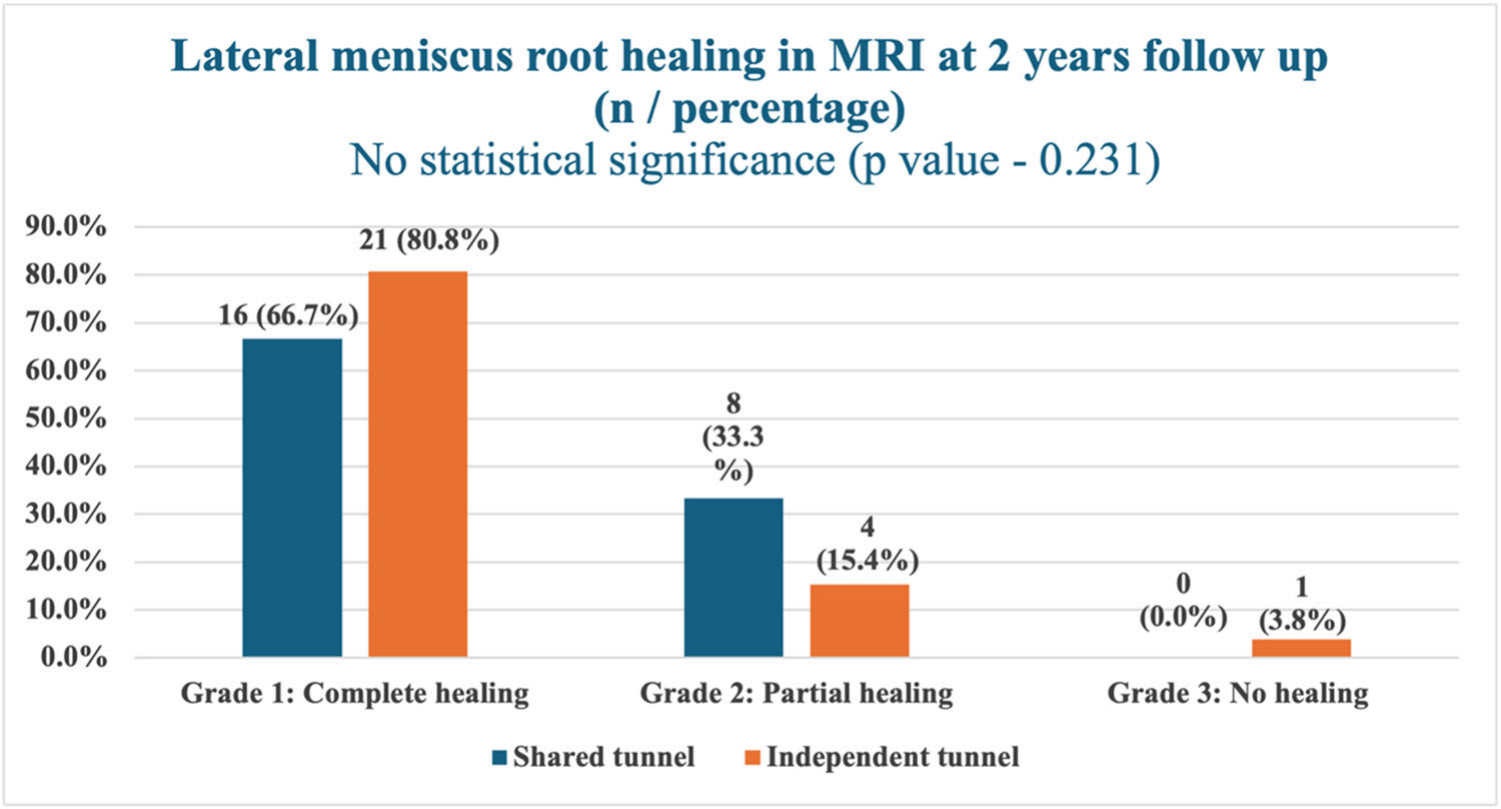
Distribution of lateral meniscus posterior root healing grades on MRI at 2‐year follow‐up in the shared tunnel (*n* = 24) and independent tunnel (*n* = 26) groups. Bars represent the percentage of patients in each healing category, with values displayed as number of patients (percentage). Comparison between groups demonstrated no statistically significant difference in healing distribution (*p* = 0.231). MRI, magnetic resonance imaging.

### Return to play

Return to non‐contact sports was achieved at a mean of 6.1 ± 0.9 months in the shared tunnel group and 6.3 ± 1.0 months in the independent tunnel group, with no significant difference between groups (*p* = 0.42). Return to full contact or pivoting sports occurred at a mean of 8.4 ± 1.1 and 8.6 ± 1.2 months, respectively, with no statistically significant difference (*p* = 0.51). At final follow‐up, return to pre‐injury level of sports participation was observed in 21 of 24 patients (87.5%) in the shared tunnel group and 23 of 26 patients (88.5%) in the independent tunnel group, with no significant difference between groups (*p* = 0.91).

### Complications and revisions

A single patient in the independent tunnel group demonstrated failed LMPR healing on MRI at the 2 year follow‐up; however, the patient remained asymptomatic and no clinical complications were observed. No intraoperative or postoperative complications were observed. No revision surgeries were required in either group during the minimum 2‐year follow‐up period, resulting in a revision rate of 0% in both groups.

## DISCUSSION

The main findings of this study were that LMPR repair performed using a shared ACL tibial tunnel resulted in clinical, patient‐reported and MRI outcomes comparable to those achieved with an independent transtibial pullout technique at 2 years of follow‐up. Additionally, the shared tunnel technique was associated with a significantly shorter operative time.

A fundamental anatomical difference between the two techniques evaluated in this study relates to the location of the tibial tunnel used for LMPR fixation. In the independent transtibial pullout technique, the tibial tunnel is drilled directly at the anatomic LMPR footprint, whereas in the shared tunnel technique, the tibial tunnel is created at the ACL tibial footprint, which is located approximately 5–6 mm anterior to the native LMPR footprint. Anatomical studies examining the spatial relationship between these footprints have demonstrated a close proximity between the LMPR and ACL tibial tunnels, particularly when both are drilled from the anteromedial tibial surface, thereby increasing the risk of tunnel convergence when independent tunnels are used [2].

Although biomechanical investigations have shown that anatomically placed tibial tunnels for LMPR repair may better restore native contact pressures and hoop stresses [18], the present study, consistent with previously published clinical series—demonstrated comparable functional and radiological outcomes between shared and independent tunnel techniques [19, 20]. This apparent discrepancy can be explained by the fact that, in the shared tunnel technique, the LMPR is anatomically reduced and stabilized at its native insertion site using transtibial sutures, despite the tibial suture exit point being non‐anatomic. Thus, while the tunnel trajectory differs between techniques, the final position of the repaired meniscus remains anatomical. These findings suggest that accurate meniscal reduction and stable fixation at the native footprint may be more critical determinants of clinical outcome than the precise location of the tibial tunnel itself, thereby explaining the comparable outcomes observed despite the non‐anatomic tibial tunnel position in the shared tunnel technique.

Zhou et al. [20] conducted a retrospective study evaluating patients who underwent ACL reconstruction using STG graft combined with LMPR repair. Their surgical technique was similar to that used in the present study, except that additional extracortical suspensory fixation was employed for the shared tunnel technique. In contrast, stable fixation in the present study was achieved using interference screw fixation alone, thereby avoiding the use of an additional implant and reducing procedural cost and operative time. Clinical, radiological and arthroscopic outcomes of LMPR repair performed using a shared ACL tibial tunnel and an independent transtibial pullout technique were compared. Comparable pivot‐shift grades, IKDC scores, Lysholm scores and Tegner activity scores were reported between the two groups. A significantly shorter operative time was observed in the shared tunnel group (98.3 ± 11.1 min) compared with the independent tunnel group (127.9 ± 17.5 min; *p* = 0.001), findings that were consistent with those of the present study (73.50 ± 8.51 min vs. 97.00 ± 8.39 min; *p* < 0.001). Comparable clinical outcomes and meniscal healing on MRI at 2 years of follow‐up were also reported. Although a significantly greater reduction in LME was observed in the shared tunnel group (2.41 ± 0.61 mm) compared with the independent tunnel group (1.59 ± 1.35 mm; *p* = 0.014), a similar trend toward greater LME reduction in the shared tunnel group (2.14 ± 0.35 mm) was observed in the present study when compared with the independent tunnel group (2.01 ± 0.35 mm), without reaching statistical significance (*p* = 0.181).

Okazaki et al. [13] performed a retrospective analysis of 17 patients who underwent double‐bundle ACL reconstruction with concomitant LMPR repair. Among these patients, transtibial pullout repair through the posterolateral bundle tibial tunnel was performed in nine cases, while inside‐out or all‐inside LMPR repair was performed in eight cases. Transtibial pullout repair was found to result in significantly greater reduction in lateral meniscal extrusion compared with other surgical techniques (*p* = 0.047), thereby contributing to improved restoration of hoop tension.

Pan et al. [15] evaluated the shared ACL tibial tunnel technique for LMPR repair performed in conjunction with ACL reconstruction and compared outcomes with those of a conservatively managed control group. At final follow‐up, significant improvements in functional outcomes were demonstrated in the shared tunnel group, with Lysholm scores improving from 59.03 ± 19.17 preoperatively to 92.34 ± 6.32 and IKDC scores improving from 62.08 ± 19.21 to 90.06 ± 8.59. Comparable improvements in clinical outcome scores were also observed in the shared tunnel group in the present study. Although comparable improvements in functional outcome scores were reported in the conservatively managed group, a lower tendency toward the development of osteoarthritis was observed in the surgically treated group.

Shekhar et al. [17] conducted a prospective study of 25 patients with combined ACL and LMPR tears. Combined LMPR repair using an independent transtibial tunnel with ACL reconstruction was performed in 23 patients, while side‐to‐side LMPR repair with ACL reconstruction was performed in two patients. Significant postoperative improvements were observed in all clinical outcome measures, with IKDC scores improving from 47.6 ± 9.5 preoperatively to 81.8 ± 11.5 at follow‐up and Lysholm scores improving from 49.0 ± 11.5 to 88.8 ± 7.6 (*p* < 0.001). These improvements were comparable to those observed in the independent tunnel group in the present study (*p* < 0.001).

In a meta‐analysis conducted by Zheng et al. [19], 92 patients underwent transtibial pullout suture repair for LMPR tears, including 56 patients treated using a shared ACL tibial tunnel technique and 36 patients treated using an independent transtibial pullout technique. Substantial improvements in clinical outcomes were demonstrated in both groups. Specifically, mean Lysholm scores improved from 53.1 preoperatively to 89.5 at follow‐up, while mean IKDC scores improved from 62.0 to 86.6. The significant improvements observed in both groups in the present study (*p* < 0.001) were consistent with these findings (Table 2).

This study has several limitations. First, the retrospective design may introduce selection bias. Second, all procedures were performed by a single surgeon. Third, LMPR tear patterns were not subclassified or analysed separately, which may influence healing potential and extrusion behaviour. Fourth, second‐look arthroscopy was not performed to confirm MRI‐based healing assessments. Finally, although the shared tunnel technique results in a non‐anatomic tibial suture exit point, comparable clinical and radiological outcomes were observed in the present study. However, the biomechanical consequences of this non‐anatomic suture trajectory were not directly evaluated and longer‐term clinical studies combined with dedicated biomechanical analyses are required to better define its implications.

## CONCLUSION

LMPR repair performed using a shared ACL tibial tunnel resulted in clinical, patient‐reported and MRI outcomes comparable to those achieved with an independent transtibial pullout technique at a minimum follow‐up of 2 years. The shared tunnel technique was associated with a significantly shorter operative time, without compromising knee stability, functional recovery, meniscal healing or LME.

By eliminating the need for an additional tibial tunnel, the shared tunnel technique preserves bone stock, avoids the risk of tunnel convergence and obviates the requirement for an additional implant, thereby reducing procedural complexity and cost. These findings support the shared ACL tibial tunnel technique as a safe, efficient and effective option for LMPR repair when performed in conjunction with arthroscopic ACL reconstruction.

## AUTHOR CONTRIBUTIONS

Conceptualization and methodology of the study were done by Ashwanth Narayan Balashanmugam and Sanjay Ayyakkannu Kasthuriranganathan. Data curation, manuscript original draft preparation and visualization were done by Ashwanth Narayan Balashanmugam. Manuscript review and editing was done by Ashwanth Narayan Balashanmugam and Sanjay Ayyakkannu Kasthuriranganathan. Investigation and validation was done by Ashwanth Narayan Balashanmugam and Praveen Ravi. Software and formal analysis were taken care of by Vignesh Murali. Project was supervised by Sanjay Ayyakkannu Kasthuriranganathan. The project was administered by Suresh Perumal. Resources were provided by Arumugam Sivaraman.

## CONFLICT OF INTEREST STATEMENT

The authors declare no conflicts of interest.

## ETHICS STATEMENT

This study was approved by the Institutional Research Ethics Committee of Sri Ramachandra Institute for Higher Education and Research, Porur (REF: CSP‐MED/22/FEB/74/24). All procedures performed were in accordance with the ethical principles mentioned in the Declaration of Helsinki (2013). Informed consent was obtained from all the participants of the study.

## Data Availability

The data that support the findings of this study are available on request from the corresponding author. The data are not publicly available due to privacy or ethical restrictions.
